# Magnetite-Assisted Capture Affinity, Concentration Dependence, and Magnetic Extraction Rate of *Bacillus cereus*

**DOI:** 10.3390/microorganisms13061176

**Published:** 2025-05-22

**Authors:** Gouri Nilakshika Atapattu, Michelle Giltrap, Furong Tian

**Affiliations:** 1 School of Food Science & Environmental Health, City Campus, Technological University Dublin, Grangegorman, D07 ADY7 Dublin, Ireland; 2 Sustainability & Health Research Hub, Technological University Dublin, Grangegorman, D07 ADY7 Dublin, Ireland; 3 Radiation and Environmental Science Centre, Physical to Life Sciences Research Hub, Technological University Dublin, City Campus, Camden Row, D02 HW71 Dublin, Ireland; 4 Nanolab Research Centre, Physical to Life Sciences Research Hub, Technological University Dublin, Camden Row, D08 CKP1 Dublin, Ireland

**Keywords:** iron oxide, nanoparticle, bacterial capture, *Bacillus*, bacterial viability, interactions

## Abstract

The interactions between magnetic nanoparticles (MNP) and bio-surfaces have impacted key industries such as food, biomedicine, water purification, and agriculture. Bacteria, with their diverse bio-surfaces, offer potential for such interactions. Yet, there is a paucity of research interpreting the dynamics behind bacteria–nanoparticle interactions. Advancing this knowledge could improve the industrial applications. Current research gaps include information about the magnetic nanoparticle-assisted concentration dependence of *Bacillus cereus* and determination of the rate of bacterial extraction by MNPs such as iron oxide nanoparticles (IONPs). Using magnetic IONPs as the choice of MNP, this study aimed to investigate in vitro the interactions between model bacteria and IONPs, leveraging the bacterial magnetising property. IONPs were synthesised by co-precipitation and characterised. Magnetic capture efficiency was reported for four model bacteria (*Bacillus cereus*, *Escherichia coli*, *Staphylococcus aureus*, and *Salmonella typhimurium*). The effect of particle concentration on the viability of *Bacillus cereus* and the rate of magnetic extraction of *Bacillus cereus* were evaluated. *Bacillus cereus* had the most robust interaction with IONP (90.34%). While the magnetic extraction was time-dependent, the average rate of magnetic extraction for *Bacillus cereus* was calculated as 3.617 CFU mL^−1^/min. Growth inhibition at 1.0, 2.0, and 4.0 mg mL^−1^ of IONP was significant. Magnetic capture results indicated that members of the Bacillus genus screened for plant growth-promoting traits may be suitable to combine with IONPs for future land application.

## 1. Introduction

Nanotechnology is gaining significant attraction among researchers [1] due to its wide application in various domains, such as biomedicine [2], food science [3], water treatment [4], agriculture [5], electronics [5], and mechanics [6]. Currently, exploiting interactions between magnetic nanoparticles (MNPs) and bacteria has benefitted biomedicine for drug delivery and gene delivery [7], the food industry for biosensors that detect food-borne pathogens and microbial toxins [8], and in water purification for pathogen removal [9]. Microbial inoculants coupled with nanoparticles have been more effective in the removal of heavy metals and organic and inorganic pollutants (bioremediation processes) [10]. Silver or titanium coated with magnetic nanoparticles are considered promising antibacterial agents against bacterial pathogens such as *E. coli*, *S. aureus*, and *S. epidermidis* [11,12]. The agricultural sector has also started employing MNPs to improve crop production. Some nanoparticle types, such as iron oxide, zinc oxide, copper oxide, and manganese-based compounds, are considered to have nanofertiliser properties [13]. Currently, it is suggested that when nanofertilisers are encapsulated with beneficial bacteria such as *Bacillus* spp., *Azotobacter*, and *Rhizobium*, crop productivity could be significantly and sustainably enhanced [14]. Out of the several bacterial models tested for their interactions with magnetic nanoparticles in the literature thus far, *Bacillus cereus* has not been widely examined for the concentration-dependent behaviour or toxicity exerted by magnetic nanoparticles such as Fe_3_O_4_. Moreover, capture affinity (capture efficiencies) has been mostly reported for ultra-low concentrations of magnetic nanoparticles. In addition, the literature has very limited comparisons of capture affinity among bacterial models such as *Bacillus cereus*, *Escherichia coli*, *Staphylococcus aureus*, and *Salmonella typhimurium*. Furthermore, no evidence has been documented to elucidate the role of time in the magnetic capture of bacteria, especially *Bacillus cereus*. Magnetic extraction of bacteria could be either time-dependent or time-independent. Hence, it is a topic worthy of investigation as it is an important parameter for applying nano–bio interactions in an industry setting. It determines the rate of bacterial removal or extraction, which eventually addresses the efficiency.

Towards this end, several magnetic nanoparticle-bacteria based studies have been reported. Magnetic nanoparticles and bacteria interact via electrostatic interactions (such as Van der Waals interactions) and hydrophobic interactions [15]. Based on this concept, capture affinity/capture efficiency can be reflected for different bacterial models. For example, ultra-low concentrations of positively charged Fe_3_O_4_ nanoparticles (5–100 μg/mL) were found to capture *Escherichia coli* cells efficiently [16]. Similarly, Quintana-Sánchez et al. (2022) employed cationic-modified magnetic nanoparticles (62.5–2000 μg/mL) to study the capture efficiency of *Escherichia coli* and *Staphylococcus aureus* cells [17]. *Listeria monocytogenes* and *Staphylococcus aureus* were investigated for capture affinity for chitosan-coated magnetic nanoparticles [18]. Utilising glycan-coated magnetic nanoparticles was also successful in inducing affinity towards bacteria such as *Salmonella Enteritidis* and *Escherichia coli* O157:H7 [19]. In another instance, marine bacterial strains such as *Bacillus velezensis* and *Bacillus subtilis* were successfully extracted by bare Fe_3_O_4_ magnetic beads with an 80% capture efficiency [20]. However, it was reported that when Fe_3_O_4_ nanoparticles (100–600 mg/mL) were modified, bacterial capture of *Bacillus subtilis* and *Escherichia coli* was enhanced. Parallelly, in the presence of functionalised NP doses such as 100–600 mg/mL, inactivation of bacterial cells was also observed [21].

The viability of cells may alter as a result of bacteria exposure to magnetic nanoparticles with some studies claiming that IONPs in general display moderate antibacterial activity [22]. However, when reporting the viability or antimicrobial activity of IONPs for bacteria, the concentration and type of bacteria must be noted. Ranmadugala et al. (2017) discovered that the viability of *Bacillus subtilis* decreased in the presence of iron oxide nanoparticles above 300 μg/mL [23]. Abou-Dobara et al. (2024) discovered that bacteria such as *B. cereus* ATCC6633, *S. aureus* ATCC25923, and *E. coli* displayed concentration-dependent inhibition against Fe_2_O_3_ NPs with a concentration range of 5–50 μg/mL [24]. Likewise, Fe_3_O_4_ NPs (31.3–1000 μg/mL) triggered a concentration-dependent inhibition against bacteria such as *S. aureus* (ATCC 29213) and *S. typhimurium* (ATCC 29629). However, this inhibiting effect from Fe_3_O_4_ NPs was significantly lower compared to silver-coated Fe_3_O_4_ NPs [25]. In another study, Fe_3_O_4_ nanoparticles (50, 100, and 150 μg/mL) were able to inhibit *S. aureus*, *E. coli*, and *B. subtilis* [26]. Although IONP-assisted inhibition zones were investigated for bacteria, the concentration-dependent behaviour has not been adequately relayed in the literature. Moreover, in terms of bacterial models, *Bacillus subtilis*, *Staphylococcus aureus*, and *E. coli* have been exploited widely with several magnetic nanoparticles [27,28,29,30,31]. Nevertheless, *Bacillus cereus* (ATCC 11778), an industrially important organism, has not quite been described in the literature with regard to its concentration-dependent behaviour in relation to a Fe_3_O_4_ NPs concentration range such as 100–500 μg/mL warranting future work in this area.

Therefore, to fill the above research gaps, our study aimed to investigate in vitro interactions between bacteria and citrate-stabilised magnetic iron oxide nanoparticles (IONPs) with an average diameter of 16 nm using the bacterial magnetisation property. In this study, the affinity of bacteria to IONPs was deduced using bacterial capture activity (%) measurements for four different bacteria (*Bacillus cereus*, *Escherichia coli*, *Staphylococcus aureus*, and *Salmonella typhimurium*). In addition, a comparison was made among the above bacteria, as previous studies have not performed capture studies with this many bacterial strains. Moreover, prior studies have not considered bacteria (*Bacillus cereus* especially) and magnetic iron oxide (Fe_3_O_4_) NP interactions with an emphasis on bacterial magnetisation with time as a factor. To our knowledge, this is the first time that the effect of time on forming a bacteria–NP system has been elucidated. Considering this, our study took an initiative to fulfil the following aims: (i) to examine the magnetic capture efficiency of the model bacteria *Bacillus cereus*, *Staphylococcus aureus*, *E. coli*, and *Salmonella typhimurium* using IONPs, (ii) to examine how IONP concentration affects the viability of bacteria, and (iii) to investigate the impact of time on forming a *Bacillus cereus*–NP system and magnetic extraction.

The findings reported for a particular magnetic nanoparticle of a specific shape or size may not guarantee identical effects on bacteria when utilising a different size of the same type of magnetic nanoparticle [32,33,34]. Factors such as synthesis, shape, size, crystalline structure, and the stabiliser that is used in nanoparticle preparation can lead to different results and conclusions regarding cytotoxicity and affinity. In this context, understanding bacteria–magnetic nanoparticles interactions could benefit not only biomedicine but also agriculture as well, especially when combining biofertilisers (live-beneficial bacteria) and nanoparticles to be used in crops.

## 2. Materials and Methods

### 2.1. Materials

Iron (III) chloride, sodium hydroxide, citrate, hydrochloride acid, and ethanol were supplied by Sigma Aldrich (Darmstadt, Germany). Microbiological culture media, e.g., Mueller–Hinton broth (MHB) and Mueller–Hinton agar (MHA), were obtained from Sigma Aldrich (Darmstadt, Germany), while cation-adjusted Mueller–Hinton broth was purchased from Merck KGaA, Darmstadt, Germany.

Nutrient broth (NB) and tryptic soy agar (TSA) were supplied by Condalab (Madrid, Spain).

*Bacillus cereus* (ATCC 11778), *Escherichia coli* (ATCC 25922), *Staphylococcus aureus* (ATCC 25923), and *Salmonella typhimurium* (ATCC 14028) strains were provided by the School of Food Sciences and Environmental Health, TU Dubin (Dublin, Ireland).

The neodymium disc magnet with an N35 grade was used for magnetic extraction. According to the manufacturer, the disc magnets consisted of neodymium-iron-boron. These disc magnets were arranged in four columns, in a 2 × 2 square grid against each other. Each column consisted of four discs. The dimensions of a single disc were 5 mm in thickness and 5 mm in diameter. The direction of the magnetisation was axial. The calculated optimal magnetic field strength was 2.32 T. All the magnetic extraction steps were conducted at room temperature unless otherwise stated.

### 2.2. Synthesis and Characterisation of IONPs (Fe_3_O_4_)

The magnetic iron oxide (Fe_3_O_4_) nanoparticle was synthesised and characterised by the method described in [35]. Briefly, ammonium chloride (30 g) was dissolved in ultrapure water (150 mL), followed by degassing under argon. Freshly prepared ammonia solution (25%, 40 mL) was added into it. The solution was heated up to 90 °C under argon under mechanically stirring. Iron (II) chloride (0.86 g) and iron (III) chloride (2.33 g) were dissolved in 25 mL of ultrapure water. Iron solution was added dropwise to the ammonia solution at 90 °C under continuously stirring. The solution was further kept at 90 °C for 2 h under argon. The product was washed with water and methanol. Washed IONPs were stabilized by mixing with 0.1 g/mL of citric acid solution and stirred for 3 h at 80 °C. The solution was cooled. The product was re-washed with de-ionised water and dried. The dried product was used for characterisation. Particle’s hydrodynamic diameter was determined by the Zetasizer (Malvern Instruments, Worcestershire, UK). TEM images were captured by a JEOL 2100 Lab TEM (JEOL Ltd., Tokyo, Japan) equipped with a LaB6 electron source (200 kV). X-ray powder diffraction was performed using a Bruker D2 Phaser second-generation pXRD (Bruker Ltd., Billerica, MA, USA) at a wavelength of 0.154 nm.

### 2.3. Bacterial Capture/Affinity Studies with IONPs

**Figure 1:**
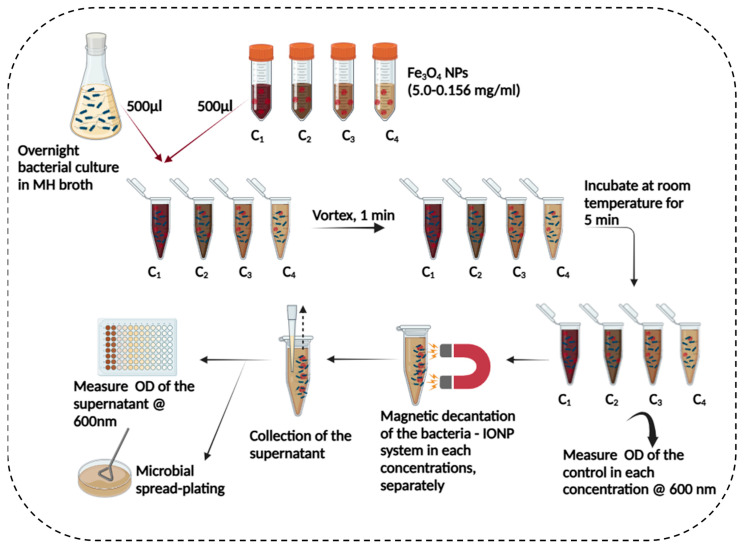
Schematic diagram of assessing the bacterial capture (magnetic capture) for a concentration gradient of iron oxide nanoparticles. C_1_−C_4_ indicates the concentration gradient of iron oxide nanoparticles. The figure was created by Bio Render.

The magnetic capture of bacteria was performed according to a previous protocol stated in [17] with slight modifications. The bacterial cultures *Bacillus cereus*, *Escherichia coli*, *Staphylococcus aureus*, and *Salmonella typhimurium* were purified three consecutive times by streak isolation followed by Gram staining and light microscopic observation as described in [36]. A single colony from each bacteria was inoculated into a sterile nutrient broth (50 mL) in an Erlenmeyer flask. It was incubated for 18–24 h at 37 °C in a shaking incubator (LABWIT, ZWYR-D2402, Shanghai, China) at 150 rpm. Log-phase cultures (10^6^–10^7^ CFU mL^−1^) of each bacterial suspension were adjusted by a UV–visible spectrophotometer at a wavelength of 600 nm (HACH LANGE GmbH, Berlin, Germany). Suspensions of IONP (Fe_3_O_4_ @ citrate) were prepared in the range of 5.0 to 0.156 mg mL^−1^. They were sterilised in an ultraviolet (UV) germicidal chamber (BioShift^®^, Shanghai, China) for 15 min. Then, 500 μL aliquots of each suspension were added into bacterial cultures (500 μL) separately. These suspension mixtures were vortexed (DAIHAN Scientific Co.,Ltd, Wonju, South Korea) for 1 min and incubated at room temperature for another 10 min. The magnetised bacterial system was captured by magnetic decantation for 10 min using a Neodymium magnet (diameter: 5 mm; thickness: 5 mm) unless otherwise stated. The supernatant was pipetted out, and optical density (OD) was measured in the supernatant and the initial solution at 600 nm. The protocol is illustrated in Figure 1. The percentage of bacterial capture was determined by the following equation.

Percentage bacterial capture = (OD_initial_ − OD_supernatant_)/OD_initial_ × 100 (1)

### 2.4. In Vitro Evaluation of IONPs on the Viability of Bacillus cereus

#### 2.4.1. Comparison of Magnetic Extraction of *Bacillus cereus* with Controls

The bacterial log-phase cultures (100 μL aliquots) were transferred into three separate sterile Eppendorf tubes. Three different concentrations of synthesised iron oxide nanoparticles were prepared (2.5, 3.33, and 3.75 mg mL^−1^) for the initial assessment and added to the bacterial working cultures. The positive control (*B. cereus* with IONPs but not subjected to magnetic extraction) and negative control (*B. cereus* with sterile de-ionised water but without IONPs) were set up simultaneously. Each tube was vortexed for 1 min and incubated in a shaking incubator (400 rpm) at 37 °C for 1 h. An external magnetic field was applied to the walls of the Eppendorf tubes using the Neodymium magnet to extract the bacterial cells attached to the IONPs. The supernatant was pipetted out and serially diluted. The dilutions were pour-plated on nutrient agar in triplicates. The plates were incubated at 37 °C for 24 h, and colony establishment was observed.

#### 2.4.2. Colony-Forming Unit (CFU) Assay

A purified single colony of *Bacillus cereus* (as described in Section 2.3) was transferred into a sterile MH broth (25 mL) in a 100 mL Erlenmeyer conical flask. The suspension was incubated for 18 h at 37 °C in a shaking incubator (LABWIT, ZWYR-D2402, Shanghai, China) at 150 rpm. The log-phase culture of *B. cereus* was obtained in sterile Mueller–Hinton broth.

A stock solution of iron oxide nanoparticles was sterilised by UV irradiation (BioShift^®^, China) for 15 min. It was used for the following experiment. A concentration range (4.0–0.125 mg mL^−1^) of magnetite iron oxide nanoparticles was prepared by two-fold dilutions. *Bacillus cereus* in MH broth was added to tubes with different IONP concentrations. Parallelly, a control was set up by adding *Bacillus cereus* in Mueller–Hinton broth with de-ionised water. The tubes were incubated at 37 °C for 90 min. After the incubation period, the tubes were mixed using sterile pipettes. IONPs with *Bacillus cereus* in each tube were serially diluted (10^−1^–10^−5^) in sterile phosphate-buffered saline solution. The control was serially diluted in (10^−1^–10^−5^) in sterile phosphate-buffered saline solution. Each dilution was spread-plated on sterile Muller–Hinton agar. The plates were incubated in an inverted position at 37 °C for 18 h. Colonies were counted on each plate. Viable bacteria CFU mL^−1^ was calculated for each IONP concentration and the control separately.

#### 2.4.3. Growth Kinetics for *Bacillus cereus*

Bacterial growth kinetics were studied according to the protocol described in (Arakha et al., 2015 [37]) with some modifications. The *Bacillus cereus* log-phase culture was obtained in cation-adjusted MH broth as described in Section 2.4.2. A stock solution of IONPs was sterilised by UV irradiation for 15 min. Two-fold dilutions of IONPs (50 μL) were prepared in a sterile 96-well plate. *Bacillus cereus* in cation-adjusted MH broth (50 μL) was added to the same wells containing nanoparticles. A positive control was set up by adding sterile de-ionised water (50 μL) to two-fold diluted iron oxide nanoparticles in a separate row. A negative control was set up by adding sterile de-ionised water (50 μL) to *B. cereus* in cation-adjusted MH broth (50 μL). The 96-well plate was incubated at 37 °C for 18 h. OD at 600 nm was measured and recorded using the spectrophotometer (HACH LANGE GmbH, Germany) at every hour for 18 h. OD_(600nm)_ values were used to construct the growth curve for *Bacillus cereus*.

#### 2.4.4. Antimicrobial Activity

A standard well-diffusion test was performed to evaluate the antibacterial activity of IONPs against bacterial pathogens. Gram-positive (*Bacillus cereus* and *Staphylococcus aureus*) and Gram-negative (*E. coli* and *Salmonella typhi*) strains were used for this test. The log-phase broth cultures of pathogenic bacteria were evenly swabbed on Mueller–Hinton agar plates. A sterile cork borer was used to create wells with a diameter of 10 mm on agar plates. IONPs with a concentration range of 5.0 to 0.625 mg mL^−1^ were used to load the wells. Ampicillin (100 μg mL^−1^) and Chloramphenicol (25 μg mL^−1^) were used as the positive controls. The Petri dishes were incubated for 24 h at 37 °C. The plates were observed for inhibition zones after the incubation period.

### 2.5. Influence of Time on Magnetic Decantation of Bacillus cereus

The *B. cereus* culture (500 μL) was transferred into six sterile Eppendorf tubes. IONP solution (500 μL) was added to the Eppendorf tubes. The tubes were vortexed for a few seconds. To see the magnetic extraction of *Bacillus cereus* cells with time (t), six time points were taken at 35 s intervals (t = 0, t = 35, t = 70, t = 105, t = 140, and t = 175 s). A magnetic field was applied for the above time intervals, and the supernatant (100 μL) was transferred into a tube containing sterile MRD broth (900 μL), and serial dilutions were prepared (10^−1^–10^−7^). Separate dilution series were prepared for each time point. The dilutions were spread-plated onto TSA and were incubated at 37 °C for 24 h. The colony counts were recorded the next day. These colony counts at a given time corresponded to the supernatant’s remaining bacteria (not extracted by the magnet). The colony count at t = 0 s was referred to as the initial bacterial load. The average rate (*r*) of magnetic extraction for *Bacillus cereus* was calculated using the following equation.

Average rate of magnetic extraction (*r*) = Magnetically yielded bacteria (CFU mL^−1^)/Time (min) (2)

Likewise, we propose the average rate (*r’*) of the above bacterial load that migrated across the highest diameter (*d*) of the Eppendorf tube towards the wall under the magnetic force of the Neodymium magnet, using the following equation. The inner diameter (*d*) of the Eppendorf tube was determined using a digital vernier calliper.

Average rate (*r’*) of bacteria at a given time = Distance (highest diameter) mm/Time (min) (3)

### 2.6. Statistical Analysis

Statistical analysis was performed in Prism version 9.4.0 Graph pad software, Inc., Boston, MA, USA. Data from the magnetic capture study (bacterial capture) were normalised (0–100%) prior to the analysis. The magnetic capture/bacterial capture datasets were analysed using one-way analysis of variance (ANOVA). Multiple comparison analysis was performed using Tukey’s test unless otherwise stated. The data corresponding to concentration dependence (CFU assay) were analysed for normality using the Kolmogorov–Smirnov test and for equivalence of variances using Levene’s test. The datasets analysed had one concentration fail the normality test. With the ANOVA assumption of normality not being met, these datasets were analysed using the Kruskal–Wallis test. Multiple comparisons were analysed by Dunn’s multiple comparisons test. Error bars of figures are presented using the mean with standard error of the mean (s.e.m.) unless otherwise stated.

## 3. Results

### 3.1. Characterisation of the Fe_3_O_4_ @ Citrate Nanoparticles

The synthesised IONP sample was monodisperse and had an average hydrodynamic diameter of 52 nm (Figure S1). The morphology of the nanoparticles was determined by TEM as spherical, and the true diameter was 16 nm (Figure S2). The XRD (Figure S3) output (red line) was in accordance with Bragg’s reflection of pure magnetite (COD 900-5838). The XRD shows six different diffraction points from 2θ values between 20° to 80°. The diffraction peak at 2θ value of 35.5° recorded as the strongest, while diffraction peaks at 2θ values of 30°, 43, 53.5, 57, and 62.5 confirmed the crystalline nature of the nanoparticles (Figure S3). The pH of the colloidal suspension after stabilisation was 6.2.

### 3.2. Bacterial Capture/Affinity Studies

**Figure 2:**
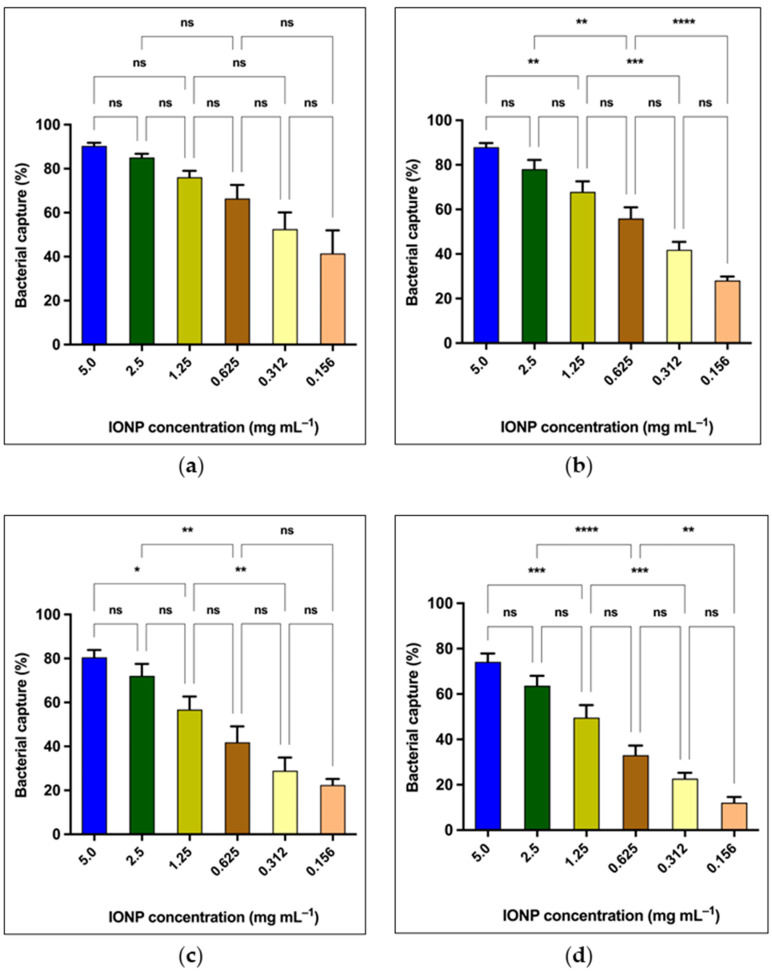
Percentage (%) bacterial capture (magnetic capture) at different concentrations of iron oxide nanoparticle for (**a**) *Bacillus cereus*; (**b**) *E. coli*; (**c**) *Salmonella typhimurium*; (**d**) *Staphylococcus aureus*. ns, not significant (*p* > 0.05); * *p* ≤ 0.05; ** *p* ≤ 0.01; *** *p* ≤ 0.001; **** *p* ≤ 0.0001. The error bars represent mean ± s.e.m., All the graphs consist of three independent biological replicates (*n* = 3).

The purpose of this experiment was to evaluate the degree of affinity of bacteria towards IONPs and to identify any significant differences between how Gram-positive and Gram-negative bacteria respond to nanoparticle capture. *Bacillus cereus* and *Staphylococcus aureus* were used as the Gram-positive strains, and *E. coli* and *Salmonella typhimurium* were used as the Gram-negative strains. According to Figure 2a, *Bacillus cereus* (Gram-positive) demonstrated the highest capture at all the concentrations employed, while *Staphylococcus aureus* showed the least potential to be captured by IONPs (Figure 2d). There was no significant difference (*p* > 0.05) between the percentage of bacterial capture at almost all the concentrations for *B. cereus* (Figure 2a). However, the rest of the bacteria, including *E. coli*, Salmonella, and *S. aureus* exhibited significant capture differences among some IONP concentrations (Figure 2b–d). At the highest concentration of IONP (5.0 mg mL^−1^), *B. cereus* illustrated a maximum capture activity of 90.34%. The percentages of capture for *E. coli*, Salmonella, and *S. aureus* were 87.98, 80.57, and 74.24%, respectively, at 5.0 mg mL^−1^ of IONP (Figure 2b–d). The survival of captured bacteria was assessed by culture inoculation. Microbial growth was observed (5.0–0.156 mg mL^−1^) in the plates containing the bacteria–IONP system.

### 3.3. Viability of Bacillus cereus Under IONP Exposure

#### 3.3.1. Magnetic Extraction of *Bacillus cereus* with Controls Using Plate Assay

**Figure 3:**
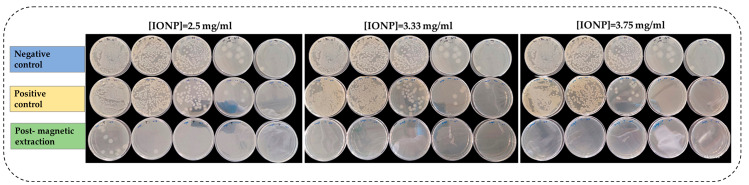
Confirming magnetic extraction of *B. cereus* cells by plate assay.

*Bacillus cereus* was chosen for this experiment as it exhibited the highest capture activity. To further examine (i) if the bacteria is magnetised by IONPs and (ii) if growth inhibition occurs in magnetised *Bacillus cereus* as the IONP concentration increases, the following experiment was conducted with two types of controls simultaneously. Negative and positive controls were previously described in Section 2.4.1. According to Figure 3, as the concentration of the IONP solution increased, the magnetic extraction of bacteria also increased. This was confirmed by the disappearance of bacterial colonies (post magnetic extraction) as the concentration increased. Magnetically extracted bacteria in each of the three different concentrations (2.5, 3.3, and 3.75 mg mL^−1^) of IONP were significantly different from each other (*p* < 0.05) (Figure S4). However, there was a slight inhibition of growth in the positive control as the concentration increased.

#### 3.3.2. CFU Assay for Viability of *B. cereus*

**Figure 4:**
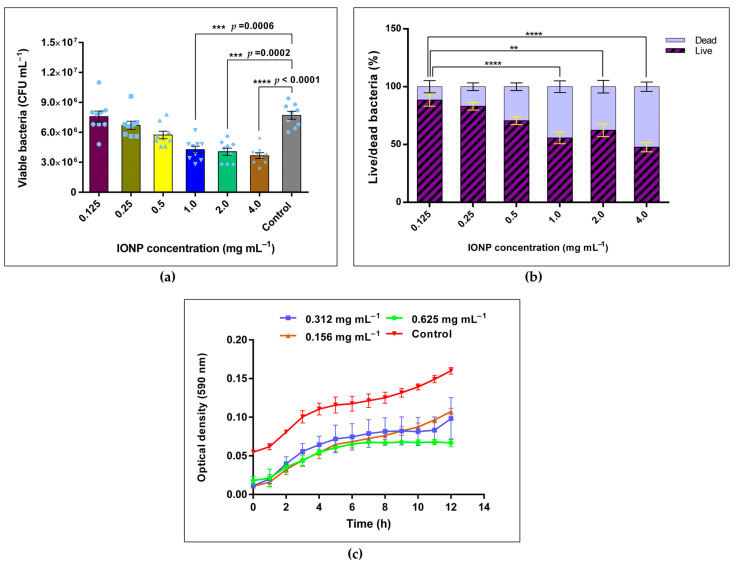
(**a**) Concentration dependence of *B. cereus* with IONPs and the viability of bacterial cells after 18 h of incubation by CFU assay; (**b**) live/dead bacterial percentage upon exposure to IONPs. ** *p* ≤ 0.01, *** *p* ≤ 0.001; **** *p* ≤ 0.0001. The error bars represent mean ± s.e.m. The graph consists of three independent biological replicates (*n* = 3). (**c**) Growth curve of *Bacillus cereus* with IONPs after 12 h of incubation. The error bars represent mean ± s.d.

Growth inhibition of *B. cereus* with IONPs was observed in the previous section. Therefore, the following experiment was conducted to quantitatively examine the degree of growth inhibition of *Bacillus cereus* for a concentration gradient of IONPs. An IONP concentration range of 0.125 to 4.0 mg mL^−1^ was used. According to Figure 4a, the number of viable *Bacillus cereus* decreased as the concentration of IONPs increased from 0.125 to 4.0 mg mL^−1^ compared to the control sample. There was a significant difference in growth between the control and the IONP-treated samples of 1.0, 2.0, and 4.0 mg mL^−1^ (*p* = 0.0006, *p* = 0.0002 and *p* < 0.0001, respectively). Growth inhibition at samples 1.0, 2.0, and 4.0 mg mL^−1^ were 44.67, 47.27, and 52.45%, respectively, compared to the control. However, growth below 1.0 mg mL^−1^ of IONP demonstrated only slight inhibition of bacteria. According to the live/dead bacterial percentage illustrated in Figure 4b, IONP concentrations such as 0.25 and 0.125 mg mL^−1^ retained more than 80% of the live bacteria. In the presence of elevated IONP concentrations (1.0, 2.0, and 4.0 mg mL^−1^), the live/dead bacterial ratio gradually decreased and reached 0.912 at 4.0 mg mL^−1^ of IONPs.

Figure 4c displays the growth kinetic curve for *Bacillus cereus* in the presence of different concentrations of IONPs. An inhibition of the growth was observed in all the studied concentrations of IONP compared to the control sample. The bacteria in 0.625 mg mL^−1^ of IONP reached the stationary phase after 7 h of incubation, which was faster than all other concentrations (0.312 and 0.156 mg mL^−1^). A tapered log phase was observed in IONP-treated samples. The agar well diffusion method revealed that nanoparticles had very little or a non-inhibitory effect (inhibition zone) for all the bacterial models.

### 3.4. Time-Dependent Magnetic Decantation of Bacillus cereus

**Figure 5:**
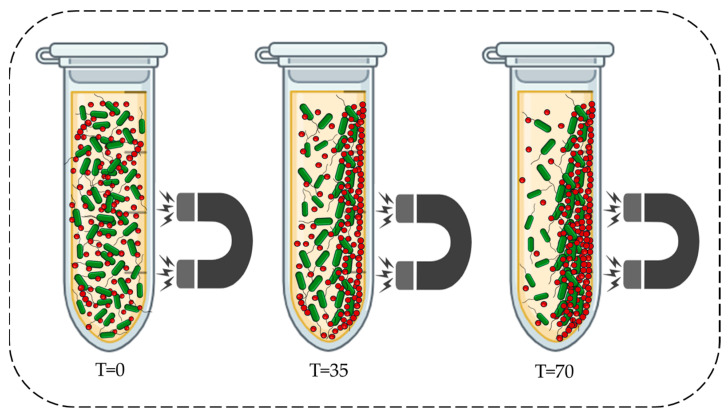
Schematic diagram: magnetic extraction of *B. cereus* at the onset (**left**), after 35 s (**middle**), and after 70 s (**right**). T indicates the time measured in seconds (s). IONPs are indicated in red colour and *B.cereus* cells are indicated in green colour. The figure was created by BioRender and Power point tool.

**Figure 6:**
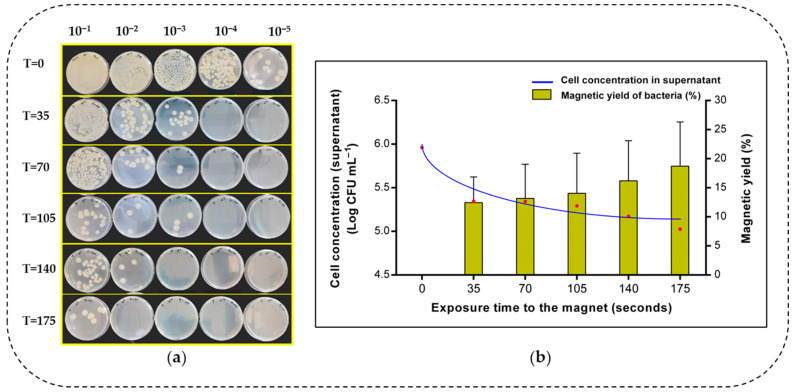
Time−dependent magnetic extraction of *B. cereus.* (**a**) Growth of *B. cereus* cells retained in the supernatant after magnetic decantation at each time interval in each serial dilution. (**b**) The graph depicts the retained bacterial cell concentration and magnetic yield vs. time of magnetic exposure. T indicates the time measured in seconds (s). CFU indicates colony-forming units. The error bars represent mean ± s.e.m. The graph consists of three independent biological replicates (*n* = 3).

Since the highest capture activity was reported from *Bacillus cereus* out of the four bacterial strains tested, it was selected for the following experiment. Magnetic extraction of bacteria could be time-dependent. However, data in this context have not been previously documented. Therefore, the influence of time on the magnetic separation of *Bacillus cereus* is examined in this section. Figure 5 illustrates how magnetic extraction was conducted at different time points. Figure 6a depicts the bacterial growth in the supernatant corresponding to each time point. Maximum growth was seen at the onset of magnetic decantation (T = 0). As the exposure time to the magnet increased, the bacteria in the supernatant decreased. This is illustrated in Figure 5, where, at onset and after 70 s of magnetic extraction, the supernatant turned clear due to the bacteria–IONP system being extracted to the magnetic field. According to Figure 6a, the amount of *B. cereus* cells (log CFU mL^−1^) that were retained in the supernatant at each time point of magnetic extraction is indicated. There was a decrease in the number of cells at the beginning as the magnetic field was applied. After the time point at 105 s, the reduction in cells in the supernatant approximately came to a steady state, where magnetic yield was approximately 20%.

Based on Figure 6b, we propose the rate (*r*) of magnetic extraction of *Bacillus cereus*, using the Equation (2).

According to the above equation and Figure 6b, the average rate of magnetic extraction for *Bacillus cereus* calculated after 175 s in our study is 3.617 CFU mL^−1^/min. Likewise, we propose the average rate (*r’*) of the above bacterial load that migrated towards the Eppendorf tube’s wall adjacent to the Neodymium magnet, using the Equation (3).

Hence, after 175 s of time, the rate of bacterial cell migration across the solution is approximately 2.995 mm/min.

## 4. Discussion

In this study, the interactions between bacteria and iron oxide nanoparticles were investigated with a focus on affinity capture, toxicity towards bacteria (Gram-positive and Gram-negative), and time-independence, which could lead to biological applications. IONP synthesis and characterisation were performed as stated in [35]. Citrate was used to stabilise the IONPs. Carboxyl groups of citrate adsorb into nanoparticle surface and increase the negative surface charge of the IONPs, creating an electrostatic repulsion among nanoparticles. This helps to prevent the aggregation of IONPs and to achieve better dispersibility in an aqueous system. DLS analysis (Figure S1) determined the average hydrodynamic diameter of the particles as 52 nm. TEM micrographs (Figure S2) revealed a monodispersed distribution of the NPs with a 16 ± 3 nm in diameter. The XRD analysis revealed indicator peaks prominent at 2 2 0, 3 1 1, 4 0 0, 4 2 2, 5 1 1, and 4 4 0 (Figure S3). This observation was supported by previous investigations that detected similar peaks at 2 2 0, 3 1 1, 4 0 0, 4 2 2, 5 1 1, and 4 4 0 for citrate-stabilised Fe_3_O_4_ NPs [38,39].

According to Figure 2, the bacterial capture percentage decreased in the following order: *Bacillus cereus > E. coli > Salmonella typhimurium > S. aureus*. Spores of *Bacillus cereus* were found to display weak paramagnetic properties due to the manganese content in the spores [40]. Therefore, it is plausible that the highest bacterial capture of *Bacillus cereus* may be aided by both paramagnetism and the magnetic IONPs bound to the cells. The mean difference of the capture percentage across different IONP concentrations for *Bacillus cereus* was not significant (Figure 2a). This implies that IONPs can capture *Bacillus* cells at any concentration in a similar pattern. Similarly, the morphological characteristics of each bacteria may affect the capture percentage. The thick peptidoglycan layer of Gram-positive bacteria contains a dense set of negatively charged polymers [41], giving the bacteria a net-negative surface charge. The surface charge of the iron oxide nanoparticles was found to be −24 ± 2 mV in our study. Therefore, the interaction between IONPs and *Bacillus cereus* was more likely driven by hydrophobic interactions, hydrogen bonding, or Van der Waals forces. Additionally, *B. cereus* cells arrange as chains, which increases the cell capture by IONPs.

As opposed to *B. cereus*, the other bacterial strains tested in our study exhibited significantly different capture percentages across the concentration gradient of IONP (Figure 2b–d). These variations may be due to the difficulty of bacterial cells in coming into close contact with IONPs in diluted concentrations, or the amount of IONPs may not be adequate to mediate an efficient magnetic capture of the cells [42]. However, this was resolved when bacterial strains were exposed to higher concentrations like 5.0–2.5 mg mL^−1^.

Comparatively, *E. coli* and *Salmonella typhi* have a thinner peptidoglycan layer compared to the Gram-positive bacteria. The net charge of the *E. coli* cell membrane was found to be less negative than some of the other bacterial strains [41], which may have affected the *E. coli* capture percentage in our study. In contrast to our study, previous work on *S. aureus* exhibited higher cell capture activity than *E. coli*. The authors stated it was due to specific carbohydrates protruding from the cell surface of *S. aureus* [43]. Therefore, factors such as secretions specific to each bacterial species may govern the bacteria–nanoparticle interaction. Chwalibog et al. (2010) also showed this via TEM images of *S. aureus* and nanoparticles [44]. Similarly, Yang et al. (2022) observed that *E. coli* secreted extracellular polymeric substances (EPS) upon exposure to silver NPs [45]. Moreover, extracellular metabolites (exopolysaccharides, proteins, and siderophores) of bacteria in the aqueous system adsorbing onto the nanoparticle surface may increase the colloidal stability of IONPs [46,47]. Likewise, it may change the surface charge of the nanoparticles. Proteins, lipids, and other cellular metabolites adsorbed around the nanoparticle surface often build a bio-corona that may affect how other particles affect bacteria, uptake by bacteria, or induce toxicity [48].

The highest bacterial capture efficiency exhibited by *Bacillus cereus* was achieved without the need to functionalise the IONP with an antibody or any other targeting molecule. Hence, it was chosen as the model organism to observe the synergistic effect of IONP concentration on viability. The low amount of bacterial growth on plates (post magnetic extraction) compared to positive and negative controls (Figure 3) confirmed that *Bacillus cereus* was magnetised by IONPs and separated from the solution during the magnetic extraction process. Therefore, colony growth post magnetic extraction was significantly lower than the controls (Figure S4). However, positive controls only showed slight inhibition of growth as the IONP concentration increased, primarily due to a large number of bacterial cells remaining in the solution, as the cells were not magnetically extracted. Moreover, the thick peptidoglycan layer of *Bacillus* sp. reduces susceptibility to oxidative stress [49]. Thus, bacteria grow at even higher concentrations of IONP. But, in some instances, cells cannot avoid oxidative stress, leading to cell death [50]. This could explain the partial inhibition of growth in the positive control (Figure 3).

A CFU assay was performed to further examine the viability of *Bacillus* cells for a IONPs concentration gradient of 0.125–4.0 mg mL^−1^. The viability decreased from 0.125 to 4.0 mg mL^−1^ in a concentration-dependent manner (Figure 2b). Growth was significantly reduced at 1.0, 2.0, and 4.0 mg mL^−1^ of IONPs compared to the control. This could be due to the inhibiting effect exerted by high concentrations of IONPs. Similar observations have been reported previously, where the growth of *B. subtilis* was significantly reduced in the presence of IONPs (300 and 400 μg mL^−1^) [23]. Evidence has been found where IONPs induced the growth of *Pseudomonas aeruginosa* [51] and *B. cereus* [23]. IONPs serve as an endogenous source of iron for bacteria, leading to elevated growth levels. However, the particle size of IONPs affects the growth pattern [23]. Aside from IONPs, low concentrations of TiO_2_ nanoparticles were shown to positively induce the growth of soil microflora, whereas high concentrations of TiO_2_ were found to inhibit the growth [52].

Observations of this CFU assay led to the addition of antimicrobial studies., i.e., an agar well diffusion assay. However, no inhibition zones were detected for *B. cereus*, *E. coli*, *Salmonella typhimurium*, and *Staphylococcus aureus*. Toxicity is often noticeable in agar, which has a lower pH. However, our medium’s pH was 7.3 ± 0.1. Hence, there is a possibility of not showing an inhibition zone on an agar matrix, which was evident in the previous literature [53]. On the contrary, Ansari et al. (2017) reported that *Bacillus cereus* exhibited inhibition zones (13 and 22 mm) at 40 and 80 μg per well of IONP, respectively [54]. Not only were the values (40 and 80 μg per well) comparatively higher than what we employed in our study, but the average diameter of the nanoparticle was 24 nm, which was larger than the particle diameter of this study (16 nm). Additionally, the maximum volume of IONP inside a well was approximately 30 μg per well in our study, which might not be adequate to exert a visible cell inhibition effect on the agar.

The effect of time on the magnetic extraction of bacteria was assessed in our present study. This correlates to the speed at which bacteria bind with IONPs. The aqueous supernatant contained non magnetised bacteria. According to Figure 6a, *Bacillus cereus* cells in the supernatant gradually decreased with time. Our data confirms that magnetic separation of *Bacillus cereus* was time-dependent. The impact of time on separating a bacteria–IONP system is essential for evaluating its biological application. It is worth mentioning that the magnetic force of the neodymium magnet would affect the rate of bacterial cell migration. Moreover, the initial bacterial cell concentration, the aqueous medium, and the total volume may affect the rate of cell migration. Therefore, further studies should consider these factors when assessing the rate (*r*) of extraction of bacteria. Previous studies have only evaluated how contact time (incubation time) affects the bacterial separation efficiency and only in few instances [55].

We anticipate that this study will broaden and contribute to the knowledge about NP–bacteria interactions for future application in agricultural fields. Findings based on affinity and toxicity of nanoparticles towards bacteria are important parameters for their use for biological applications. Our future studies will include employing IONPs with closely related members of the Bacillus genus that have plant growth-promoting traits such as phosphate solubilisation, siderophore production, indole acetic acid (IAA) production, nitrogen fixation, heavy metal tolerance, etc. These plant growth-promoting *Bacillus* sp. (wild types) were isolated from Irish peatlands in our previous studies [36,56]. Their affinity/compatibility to low doses of IONPs (compared to the doses reported in this study) will be evaluated to formulate novel nano-biofertilisers for land application as a sustainable approach to improving plant growth and yield and enhancing soil quality, thereby positively impacting the environment.

## 5. Conclusions

According to our study, *Bacillus cereus* recorded the highest bacterial capture efficiency among the four bacterial models tested. During the magnetic capture/extraction, cell inhibition may have occurred to some degree. The viability of *Bacillus cereus* cells was significantly reduced above the IONP concentration of 1.0 mg mL^−1^. Our results indicate that IONP doses below 0.5 mg mL^−1^ are suitable for retaining more than 80% of the live bacteria. The higher magnetic extraction rate of *Bacillus cereus* indicates strong surface interactions between the bacteria and nanoparticles. Furthermore, the rate of magnetic extraction can be incorporated when scaling up bacterial separation processes.

## Data Availability

The original contributions presented in this study are included in the article/Supplementary Materials. Further inquiries can be directed to the corresponding author.

## Supplementary Materials

The following supporting information can be downloaded at https://www.mdpi.com/article/10.3390/microorganisms13061176/s1: Figure S1. Characterization of IONPs: DLS histogram of the Fe_3_O_4_ @ Citrate indicating their hydrodynamic magnitudes; Figure S2. TEM images of Fe_3_O_4_ @ Citrate nanoparticles; Figure S3. Characterization of IONPs: XRD spectrum of synthesised Fe_3_O_4_ @ Citrate NPs; Figure S4. Demonstration of magnetic extraction/capture of *Bacillus cereus* with positive and negative controls using plate assay; C1, C2 and C3 refer to different IONP concentrations 2.5 mg ml^−1^, 3.33 mg mL^−1^ and 3.75 mg mL^−1^ respectively; The error bars represent mean ± s.e.m. (*n*=3); * *p* ≤ 0.05; Figure S5. VSM magnetisation curve of iron oxide nanoparticles.

## Abbreviations

The following abbreviations are used in this manuscript:

MNP	Magnetic nanoparticle
IONP	Iron oxide nanoparticle
Fe_3_O_4_	Magnetite
NP	Nanoparticle
Fe_2_O_3_	Maghemite
MHB	Mueller–Hinton broth
MHA	Mueller–Hinton agar
NB	Nutrient broth
TSA	Tryptic soy agar
UV	Ultraviolet
OD	Optical density
CFU	Colony-forming units
ANOVA	One-way analysis of variance
SEM	Standard error of the mean
DI	Dispersity index
XRD	X-ray diffraction
SD	Standard deviation
TEM	Transmission electron microscopy
DLS	Dynamic light scattering
EPS	Extracellular polymeric substances
IAA	Indole acetic acid
