# Supplementary material for: Magnetite-Assisted Capture Affinity, Concentration Dependence, and Magnetic Extraction Rate of Bacillus cereus
Source: Microorganisms. 2025 May 22;13(6):1176. doi: 10.3390/microorganisms13061176 (PMC12195268; doi:10.3390/microorganisms13061176)
Supplement: Supplementary file 1 [file microorganisms-13-01176-s001.zip › microorganisms-3592365-supplementary.pdf]

Supplementary information

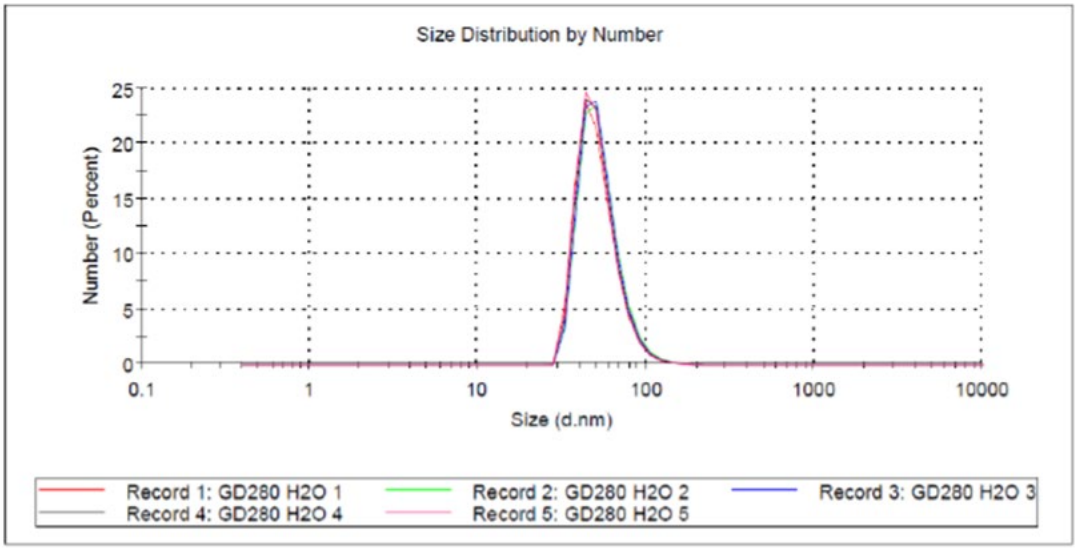

Figure S1. Characterization of IONPs: DLS histogram of the Fe_3O_4 @ Citrate indicating their hydrodynamic magnitudes

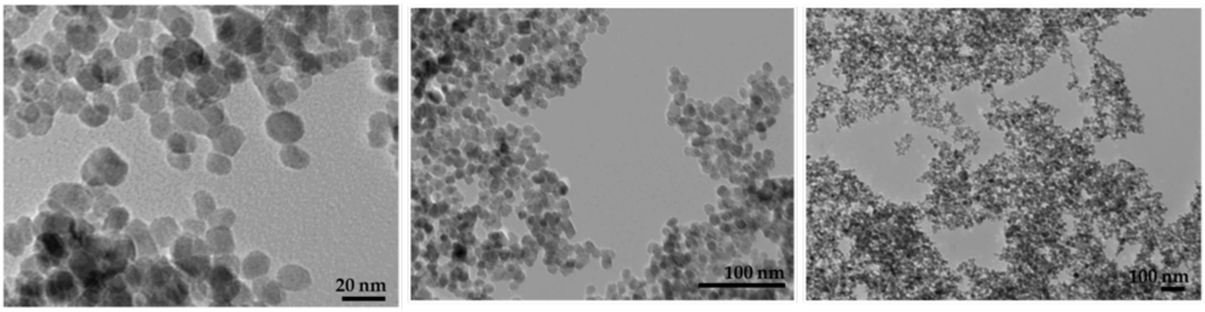

Figure S2. TEM images of Fe_3O_4 @ Citrate nanoparticles

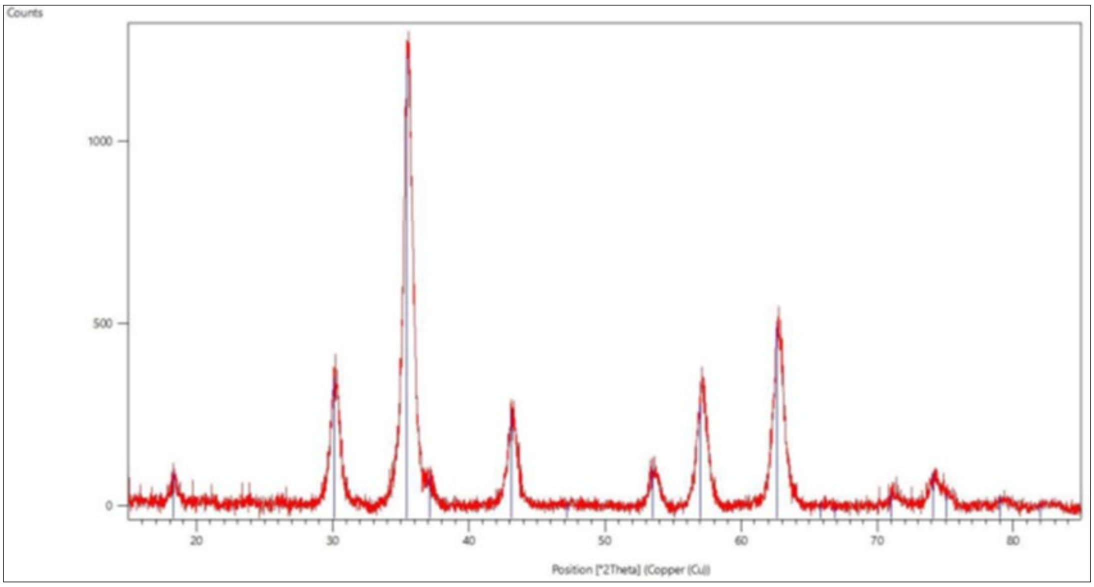

Figure S3. Characterization of IONPs : XRD spectrum of synthesised Fe_3O_4 @ Citrate NPs

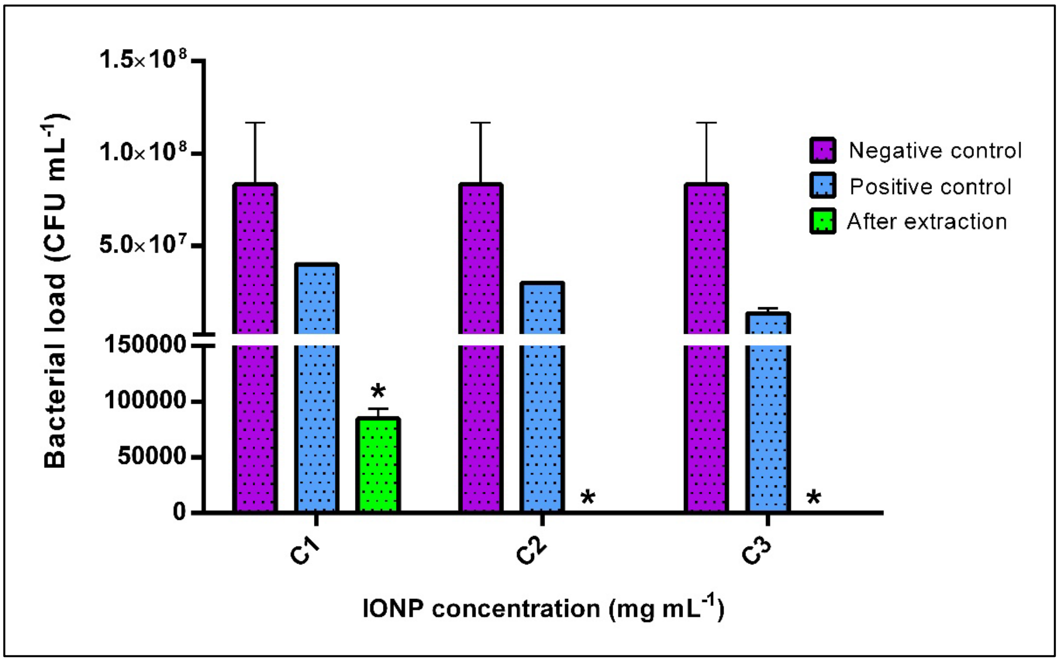

Figure S4. Demonstration of magnetic extraction/capture of *Bacillus cereus* with positive and negative controls using plate assay; C1, C2 and C3 refer to different IONP concentrations 2.5 mg ml^{-1}, 3.33 mg ml^{-1} and 3.75 mg ml^{-1} respectively; The error bars represent mean ± s.e.m. (n=3); * $p \leq 0.05$

Dimension of the magnet: Authors used a neodymium magnet with an N35 grade. The magnets are made of neodymium-iron-boron according to the manufacturer. The arrangement of the magnet was 4 vertical columns with each column having 4 neodymium disc magnets (total of 16 discs). A single disc magnet had a 5 mm thickness and a 5 mm diameter.

Therefore, the length of each column = 4 discs × 5 mm= 20 mm

The columns were arranged in a 2 × 2 square grid against each other.

Therefore, the direction of magnetization can be described as 'axially magnetized'.

The ambient temperature was the room temperature of the laboratory, which was maintained at a constant of 21°C.

Distance from the attracted particles was 1.61 mm.

Calculation of optimal magnetic field:

Using the above information, the magnetic field strength was calculated using the following equation;

$B(0)= B_r/2\ [L/\sqrt{R^2 + L^2}]$

B_r = Remanence is 1.17 T for N35 magnet

R= radius= (5 mm/2)= 2.5 mm

L= 20 mm

Since we employed 4 columns;

$B_{total} \approx 2Br.\ [L/\sqrt{R^2 + L^2}]$

Totatl field strength Btotal ≈ 2.32 T.

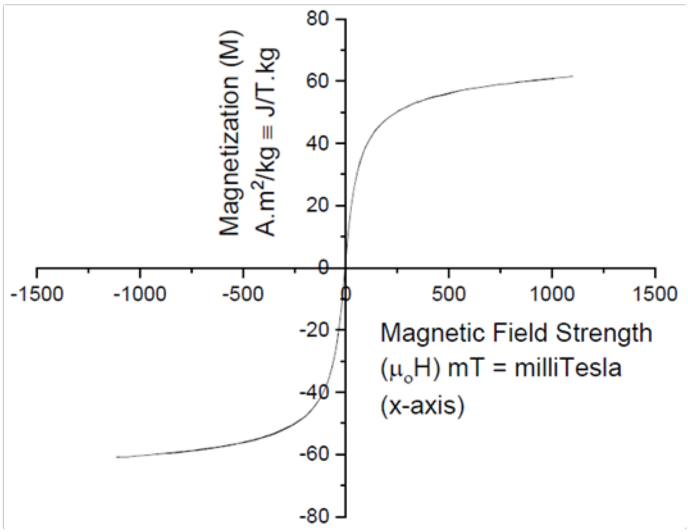

Figure S5. VSM magnetisation curve of iron oxide nanoparticles.
